# Health facility readiness to screen, diagnose and manage substance use disorders in Mbale district, Uganda

**DOI:** 10.1186/s13011-023-00570-x

**Published:** 2023-11-04

**Authors:** Harriet Aber-Odonga, Fred Nuwaha, Esther Kisaakye, Ingunn Marie S. Engebretsen, Juliet Ndimwibo Babirye

**Affiliations:** 1 School of Public Health, Makerere University College of Health Sciences, P.O Box 7072, Kampala, Uganda; 2 Centre for International Health, Department of Global Public Health and Primary Care, University of Bergen, Bergen, 7804 Norway

**Keywords:** Health facility, Readiness, Substance use, Substance use disorders, Skills, Trained personnel, Care, Treatment, Uganda

## Abstract

**Background:**

Substance use disorders (SUD) pose a significant public health problem in Uganda. Studies indicate that integrating mental health services into Primary Health Care can play a crucial role in alleviating the impact of SUD. However, despite ongoing efforts to integrate these services in Uganda, there is a lack of evidence regarding the preparedness of health facilities to effectively screen and manage SUD. Therefore, this study aimed to assess the readiness of health facilities at all levels of the health system in Mbale, Uganda, to carry out screening, diagnosis, and management of SUD.

**Methods:**

A health facility-based cross-sectional study was carried out among all the 54 facilities in Mbale district. A composite variable adapted from the WHO Service Availability and Readiness Assessment manual (2015) with 14 tracer indicators were used to measure readiness. A cut-off threshold of having at least half the criteria fulfilled (higher than the cutoff of 7) was classified as having met the readiness criteria. Descriptive analyses were performed to describe readiness scores across various facility characteristics and a linear regression model was used to identify the predictors of readiness.

**Results:**

Among all health facilities assessed, only 35% met the readiness criteria for managing Substance Use Disorders (SUD). Out of the 54 facilities, 42 (77.8%) had guidelines in place for managing SUD, but less than half, 26 (48%), reported following these guidelines. Only 8 out of 54 (14.5%) facilities had staff who had received training in the diagnosis and management of SUD within the past two years. Diagnostic tests for SUD, specifically the Uri stick, were available in the majority of facilities, (46/54, 83.6%). A higher number of clinical officers working at the health centres was associated with higher readiness scores (score coefficient 4.0,95% CI 1.5–6.5).

**Conclusions:**

In this setting, a low level of health facility readiness to provide screening, diagnosis, and management for substance use disorders was found. To improve health facility readiness for delivery of care for substance use disorders, a frequent inventory of human resources in terms of numbers, skills, and other resources are required in this resource-limited setting.

## Background

According to the World Health Organization (WHO), readiness refers to a facility’s ability to deliver services at a specified minimum standard, encompassing factors such as trained staff, necessary equipment and commodities, appropriate systems for quality and safety, and provider knowledge [1]. It has been widely recognized that integrating mental health services into primary care is a practical approach to bridging the treatment gap for mental, neurological, and substance use disorders (MNS) in low- and middle-income countries (LAMICs). Feasibility and intervention studies have provided strong evidence demonstrating the feasibility of integration [2, 3]. However, despite this consensus, there are still knowledge gaps regarding the readiness of these facilities to effectively manage substance use disorder [4].

In low-income countries, especially those in Sub-Saharan Africa, mental health conditions are often under-prioritized with suitable facilities and equipment, human resources, and infrastructure are often either unavailable or not in acceptable amounts. In Uganda for instance, general mental health care services are only available at the tertiary level [1], but the surge in substance use problems continues to afflict rural communities as well, indicating that task-shifting efforts to the primary health care level could increase substance use treatment provision, especially for the lower level facilities that often serve the rural population [2]. Uganda has one of the highest per capita alcohol consumption rates in sub-Saharan Africa and according to a recent study about 39.1% of children aged 12–24 used substances regularly [3]. This high alcohol consumption can be attributed to a complex interplay of social and environmental factors. These factors include a cultural acceptance of alcohol use, compounded by the stressors associated with health challenges such as HIV/AIDS, a history of traumatic experiences due to past wars, and elevated unemployment rates [4, 5]. As a result, documenting the capacities of these low-level facilities is critical in essentially identifying what gaps exist and how they can be bridged.

Numerous studies have focused on assessing the readiness of health facilities at both subnational and national levels. These assessments have covered a wide range of areas, including general service readiness [6], maternal and child health [6–8], family planning [9], sexually transmitted infections and HIV testing and counseling [10], tuberculosis [11], and major non-communicable diseases (NCDs) like cardiovascular disease [12], hypertension [13, 14], and chronic respiratory diseases [15]. However, there remains a critical gap in the literature regarding the empirically validated analysis of substance use disorders (SUD) readiness in low-resource settings, such as Uganda. Therefore, the primary objective of this study was to comprehensively assess the readiness of health facilities to effectively screen, diagnose, and manage SUD. Additionally, the study sought to identify key predictors of facility readiness in the specific context of low-resource settings. This research will contribute insights to address the existing knowledge gap and inform efforts to improve SUD services in resource-limited settings.

## Methods

### Study setting

The cross-sectional study was conducted in Mbale district from May 2022 to June 2022. Mbale is situated in the mid-eastern region of Uganda and has recently been divided into two major administrative units: The City and the District. The district comprises 54 health facilities, two private hospitals, and one regional referral hospital serving the eastern region. In 2020, the estimated population projection for the district was approximately 586,300 people [16]. Mbale was selected as the study location due to the significant prevalence of substance use among children and adolescents reported in the district coupled with its close proximity to the border of Uganda and Kenya makes it a target for unregulated access to cross-border smuggled alcoholic beverages [17, 18].

### Study participants

This study involved enrolling health workers who were responsible for the health centres (HC) and willing to participate. Prior to the interviews, we contacted a senior doctor or health worker at each Centre to provide a clear explanation of the study and extend an invitation to participate on behalf of their hospital or facility. Subsequently, face-to-face interviews were conducted with the participants, with each interview lasting approximately 30 min.

The participating health centres encompassed a range of levels. These included the Mbale regional referral hospital, which serves as a referral center for multiple districts in the Eastern region of Uganda. Additionally, there were four Health Centre (HC) IV level facilities, which are designated to cater to a target population of 100,000 people and provide services such as an operating theatre, inpatient care, and laboratory services. These HC IV facilities also serve as referral centers for HC III level facilities within their jurisdiction. Furthermore, the study included thirty-six HC III level facilities, which have a target population of 20,000 people and offer basic laboratory services, maternity care, and inpatient care. Lastly, thirteen HC II level facilities, which provide outpatient services and outreach programs only, were also included. These lower-level facilities are responsible for serving a target population of approximately 5,000 people.

### Eligibility

All health centres level facilities, including public, public-private, and NGO-run health centres, were considered eligible for inclusion in the study. In the district, a total of 57 units met the eligibility criteria. However, certain specialized facilities such as blood banks and HIV clinics that were not attached to health units, as well as specialized private hospitals that did not meet the eligibility criteria, were excluded from the study.

### Measurements

We operationalized readiness based on the guidelines provided in the WHO Service Availability and Readiness Assessment (SARA) reference manual [19]. We utilized fourteen specific tracer items (see Table 1). Each facility was assigned a score for each tracer item, with a score of one [1] indicating the presence of the item and a score of zero (0) indicating its absence. These scores were then summed across all fourteen tracer items to generate a total readiness score for each facility. Furthermore, we computed a binary readiness index to categorize facilities. Facilities with a total readiness score of less than half (7 out of 14) were classified as not meeting the readiness criteria, while those with a total readiness score of 7 or higher were classified as meeting the readiness criteria [20]. To assess the internal consistency of our readiness index, we calculated Cochran’s alpha, which yielded a value of 0.74, indicating acceptable reliability.

Below are the tracer items that were used to compute the readiness score.

**Table 1:** Facility readiness scores adapted from WHO SARA manual 2015

1. Guidelines for screening SUD
2. Guidelines for diagnosis SUD
3. Guidelines for managing SUD
4. Staff trained to screen for SUD
5. Staff trained to diagnose SUD
6. Staff trained to manage SUD
7. Uri stick test
8. Alcohol blood tests
9. Substance withdrawal medication (available)
10. Antipsychotic medications (available)
11. Anti-depressants (available)
12. Anti-convulsant drugs (available)
13. Private room for screening
14. Space for managing Mental health conditions

The presence of guidelines for screening, diagnosis, and management of substance use disorders was determined based on the availability to country-specific standards/guidelines or adapted guidelines, such as The Diagnostic and Statistical Manual of Mental Disorders, Fifth Edition (DSM V) [21], Alcohol use disorders identification test (AUDIT) [22], CAGE [23], CRAFFT [24], and the Uganda Clinical Guidelines for managing SUD [25]. These guidelines were typically observed in open working areas, particularly the triage area or examination rooms. Furthermore, we assessed whether the observed guidelines were routinely followed during the provision of care.

Regarding staff training, we considered a health facility to have received training if at least one staff member had undergone training in screening, diagnosis, and management of substance use disorders within the last two years while in service prior to data collection. Additionally, we inquired about the availability of at least one staff member who had received pre-service training in the management of substance use disorders.

We considered diagnostic tests for substance use disorders (SUD) to be present if a facility had the capability to use urine dipsticks and perform the test on-site. This included observing the availability of functioning equipment and necessary reagents for conducting the test. Alternatively, if blood alcohol tests were conducted at the facility, it was also considered as a presence of diagnostic testing for SUD.

We evaluated the presence of various categories of medicines commonly used in the treatment of substance use disorders. These categories included medicines for substance withdrawal, such as Benzodiazepines, Thiamine (Vitamin B1), Acamprosate, Naltrexone, and Disulfiram. Additionally, we assessed the availability of antipsychotic medicines such as Haloperidol, Risperidone, Olanzapine, Baclofen, Chlorpromazine, Haloperidol, Benzhexol, and Fluphenazine. Anti-depressants such as Fluoxetine, Imipramine, and Amitriptyline, as well as anticonvulsants including Phenobarbital, Carbamazepine, Sodium valproate, Ethosuximide, and Phenytoin, were also considered. To determine the presence of these medicines, we examined pharmacies or areas where they are routinely stored. The presence of at least one medicine from each category with a valid expiration date indicated the availability of commonly used medicines for the acute management of substance use disorders.

In addition, we assessed the availability of basic amenities at the facilities, including uninterrupted power supply, improved water source, access to adequate sanitation facilities for clients, access to a computer with email/internet access, and emergency transportation, as defined by the WHO service availability manual.

We collected data on the level of the health facility, facility ownership, and whether the facility was located in an urban or rural area. These factors were considered for the analysis.

### Data collection, management, and analysis

Face validity was ensured by having four health specialists (one psychiatrist, two general physicians, and one laboratory technician) read through the questionnaire. Input from these discussions was contributed to the relevance of the questions on the tool in this context. The tools were revised based on the pre-test and feedback to ensure that these would be comprehended and appropriate responses obtained from the respondents. The structured facility questionnaire was carefully pretested to ensure its relevance.

In-person interviews were conducted with the health professionals responsible for overseeing the facilities. These interviews were carried out by two skilled research assistants who possessed prior expertise in collecting facility data. The interviews were recorded using open data collect (ODK) software on a mobile phone. To augment the gathered information, outpatient record books from the previous month (March 2022) were thoroughly reviewed. Data on various variables, including patient attendance, screening results for substance use disorders (SUD), and referred cases, were extracted from these records. This comprehensive data collection process spanned a duration of one month, specifically between April and May 2022.

Data was downloaded from ODK in csv file format and exported to STATA software version 14 (StataCorp LLC) for analysis.

We calculated means and described using frequencies and percentages for facility characteristics, number of staff, and the SUD cases, screened, managed, and referred. At bivariable analysis, first, we compared mean scores of readiness using a t-test across all facility characteristics and basic amenities to test differences in mean readiness scores across these variables. Thereafter we carried out multivariable analysis by running a robust backward linear regression of the predictors of facility readiness scores. Linear regression was used to identify factors associated with the readiness scores of facilities to provide substance use management services. We also tested for multicollinearity across similar variables. Statistically significant response patterns were considered if a two- sided *p*-value was < 0.05.

### Ethical consideration

The project in which this study was nested had ethical approval obtained from the Regional Committees for Medical Research Ethics-South East Norway 6 March 2020, reference number 50. The study received ethical approval from the Makerere University School of Public Health Higher Degrees Research and Ethics Committee (SPH-2022-224) and the Uganda National Council of Science and Technology (HS2182ES). Prior to their participation, all study participants provided informed written consent, ensuring their voluntary involvement and protection of their rights.

## Results

Out of the surveyed facilities, a significant majority of the respondents who responded to the facility assessment, specifically 68.5% (37/54), had achieved a diploma in fields related to clinical medicine, nursing, or midwifery. However, it’s worth noting that only one individual, accounting for a 1.9% (1/54) of the respondents, held a master’s degree in the field of health services research. Furthermore, a predominant portion of the participants were male, constituting 63% (34/54), and over half of the participants held the position of facility in charge, amounting to 63% (34/ 54) as well (Table 2).

**Table 2:** Characteristics of respondents

Characteristics	Total (54)	%
**Sex**		
Female	20	37
Male	34	63
**Role in facility**		
Facility In charge	34	63
Ass. Facility in charge	4	7.4
Other senior staff	16	29.6
**Qualification**		
Master’s degree	1	1.9
Bachelor’s degree	8	14.8
Diploma	37	68.5
Certificate	8	14.8

Out of the 57 facilities in the district, 54 health facilities actively participated in the study, resulting in a response rate of 95%. The three facilities that did not participate included two private hospitals that declined and one inactive health II facility.

Among the participating facilities, there was a diverse distribution: one Regional Referral Hospital, four HC IVs, thirty-six HC IIIs, and thirteen HC IIs. The majority, 38 out of 54 facilities (70%), were government-owned, while 16 facilities (29%) were privately or NGO-owned. Geographically, approximately one-third of the facilities were located in urban areas 17/54 (32%), followed by peri-urban areas 20/54 (37%). This indicates that only 17 facilities (32%) were situated in rural areas. Regarding staff composition, all facilities reported the presence of a nurse/midwife, while only 18 facilities (33%) had a medical doctor on-site and majority of health facilities 47/54 (87%) had clinical officers. In terms of readiness to provide substance use disorder (SUD) services, the majority of facilities 38/54 (70%) claimed to possess the capability to screen, diagnose, and treat SUD.

Overall, the facilities exhibited a mean outpatient attendance of 7,452 cases (SD 539.5) in the last month, with an average of 449.2 (SD 502.1) patients being screened for SUD. When it comes to specific care provisions, a significant portion of facilities 44/54 (81.5%) offered both substance-induced withdrawal care and substance-induced depression care. However, only a small fraction 7/54 (12.9%) managed substance-induced psychosis. Nearly all facilities employed psychological treatment approaches, with individual treatment or counseling being the most prevalent 51/54 (94.4%), while group therapy was available in only 20 facilities (37%). In terms of drug treatments for SUD management, the majority of respondent for the facilities 45/54 (83.3%) reported the use of anticonvulsants, followed by vitamins 38/54 (70.4%) (Table 3).

**Table 3:** Distribution of facility characteristics, self-reported services offered and mean attendance by level of facility in Mbale district, Uganda

Characteristics and services offered	Total = 54 (%)	RRH (1)	HCIV (4)	HCIII (36)	HCII (13)
**Health facility (ownership)**					
Government	38 (70.4)	1	3	26	8
Private NGO/Mission	16 (29.6)	0	1	10	5
**Location**					
Urban	17 (31.5)	1	2	10	4
Peri urban	20 (37.0)	0	2	13	5
Rural	17 (31.5)	0	0	13	4
**Staffing levels per cadre**					
Doctor (yes)	18 (33.3)	1	4	11	2
Clin. Officer (yes)	47 (87.0)	1	4	36	6
Nurse/Midwife	54 (100)	1	4	36	13
**Service available**					
Both in and out-patient care	40 (74.1)	1	4	31	4
Only outpatient care	14 (25.9)	0	0	5	9
**Health facility self-reported ability to screen, diagnose and/or treat SUD**					
Only screen and diagnose	5 (9.3)	0	0	2	3
Screen, diagnose, and Treat	38 (70.4)	1	4	34	9
**SUD Conditions managed**					
Substance-induced withdrawals	44 (81.5)	1	3	33	7
Substance-induced psychosis	7 (12.9)	1	2	4	0
Substance-induced depression	45 (83.3)	1	3	31	10
**Type of SUD treatment offered**					
Psychological treatments	51 (94.4)	1	4	35	11
Drug treatments	52 (96.3)	1	4	36	11
Both	50 (92.6)	1	4	35	10
**Psychological treatment**					
Individual therapy	51 (94.4)	1	4	35	11
Family therapy	33 (61.1)	1	3	23	6
Group therapy	20 (37.0)	1	1	14	4
**Drug treatments**					
Benzodiazepines	17 (31.5)	1	2	13	1
Antipsychotics	14 (25.9)	1	3	9	1
Anti-anxiety drugs	18 (33.3)	1	3	13	1
Antidepressants	25 (46.3)	1	3	20	1
Mood stabilizers	13 (24.1)	1	1	10	1
Anticonvulsants	45 (83.3)	1	4	31	9
Vitamins	38 (70.4)	1	4	25	8
**Health facility average number of outpatient attendance (in the month of May 2022)**					
All cases Mean (SD) (May 2022)	7452 (539.5)	707	1531 (916.3)	809 (469.6)	358 (229.3)
SUD screened (SD) (May 2022)	449.2 (502.1)	174	752 (670.9)	499 (528.0)	197 (275.1)
SUD treated (SD) (May 2022)	4.2 (8.5)	33	12 (7.5)	4 (8.3)	0.5 (0.7)
SUD referred (SD) (May 2022)	1 (3.9)	4	3 (6)	1 (4.2)	0.8 (2.7)

Considering the WHO tracer items for readiness, the majority of participating facilities 42/54 (77.8%) had guidelines for SUD management, but only a quarter of the facilities 14/54 (25.9%) possessed guidelines for SUD screening. In terms of staff training, the majority of facilities 45/54 (83.3%) had staff who had received in-service mental health training, but only 6 facilities (11.1%) had staff trained within the last two years. Overall, only 8 facilities (14.8%) reported having staff trained specifically in the diagnosis and management of SUD.

Diagnostic tests for SUD were available in most facilities, with 46 out of 54 facilities (85.2%) having urine dipstick tests. However, only 11 facilities (20.4%) were capable of conducting blood alcohol/drug tests.

Out of the 54 facilities that participated in the study, a significant majority 49/54 (90.7%) had at least one substance withdrawal drug available. Additionally, more than half of the facilities 29/54 (53.7%) had at least one antipsychotic medication in stock. However, over two-thirds of the facilities 36/54 (66.7%) reported experiencing drug stock-outs for at least one of the recommended medicines for SUD management in the month prior to the study.

In terms of facility infrastructure, just over half of the facilities 33/54 (61.1%) reported having a private screening room, while only 20 facilities (37%) had dedicated space for managing mental health conditions (Table 4).

**Table 4:** Availability of WHO listed Readiness tracer items for the management of SUD by health unit type

Variable	*Total = 54 (%)	MRRH (1)	HCIV (4)	HCIII (36)	HCII (13)
**Guidelines**					
SUD screening guidelines are available at the health facility	14 (25.9)	1	1	11	1
SUD guidelines for diagnosis are available at the health facility	16 (29.6)	1	1	11	3
SUD Management guidelines are available	42 (77.8)	1	3	28	10
**Staff Training**					
In-service training of staff	45 (83.3)	1	3	31	10
Training in mental health in the last 2 years	6 (11.1)	0	2	4	0
Training in pediatric mental health (last 2 years)	6 (11.1)	0	2	4	0
Trained in SUD screening (yes in the last 2 years)	8 (14.8)	0	3	5	0
Trained in SUD diagnosis and management (in the last 2 years)	8 (14.8)	0	1	7	0
**Diagnostic tests**					
Uri-sticks are available	46 (85.2)	1	4	34	7
Blood tests for alcohol and drugs available at health facility	11 (20.4)	1	0	10	0
**Medicines**					
Substance withdrawal and treatment drugs	49 (90.7)	1	4	35	9
Benzodiazepines e.g.Diazepam	47 (87.0)	1	4	33	9
Vitamins e.g. Thiamine (Vitamin B1)	10 (18.5)	1	0	7	2
Acamprosate	0	0	0	0	0
Naltrexone	0	0	0	0	0
Disulfiram	0	0	0	0	0
*Antipsychotics*	29 (53.7)	1	3	20	5
Haloperidol	11 (20.4)	1	3	5	2
Risperidone	0	0	0	0	0
Olanzapine	0	0	0	0	0
Baclofen	1 (1.8)	0	0	1	0
Chlorpromazine	22 (40.7)	1	3	16	2
Haloperidol	9 (16.7)	1	2	5	1
Benzhexol	11 (20.4)	1	2	7	1
Fluphenazine	3 (5.6)	1	0	1	1
Anti-depressants	46 (85.2)	1	4	34	7
Fluoxetine	9 (16.7)	1	1	7	0
Imipramine	4 (7.4)	1	0	2	1
Amitriptyline	45 (83.3)	1	4	33	7
Anticonvulsants	41 (75.9)	1	4	30	6
Phenobarbital	30 (55.6)	0	3	24	3
Carbamazepine	29 (53.7)	1	3	23	2
Sodium valproate	1 (1.8)	1	0	0	0
Ethosuximide	0	0	0	0	0
Phenytoin	27 (50.0)	1	2	21	3
Drug stockouts in the last month (tracer drugs)	36 (66.7)	1	2	23	10
**Private room/ privacy**					
Private room for screening	33 (61.1)	1	3	20	8
Space for managing Mental health conditions	20 (37.0)	1	3	12	3

*(number of facilities given in parenthesis), counts and percentages reported

Among the 54 facilities assessed, only 19 (35.2%) met our criteria for readiness in screening, diagnosing, and managing substance use disorders. Among these facilities, more than a third (level 3 facilities) were found to meet the criteria, and there was one facility at level 2 that also met the criteria. The overall mean readiness score was 6.8 (SD 2.9), with the Regional Referral Hospital having the highest readiness scores among all the facilities (Table 5).

**Table 5:** Facility readiness and mean readiness scores by health unit type

Variable	Total = 54 (%)^a^	MRRH (1)	HCIV (4)	HCIII (36)	HCII (13)
Less than set readiness threshold (7)	35 (64.8)	0	1	22	12
More or equal to set readiness threshold (7)	19 (35.2)	1	3	14	1
Mean Facility readiness score, mean (SD)	6.9 (2.9)	11	9 (2)	7.3 (2.7)	4.5 (2.3)

^a^Unless otherwise specified

In our bivariable analysis, we observed statistically significant differences in the mean readiness scores among facilities based on the presence of doctors and clinical officers. Facilities with doctors and clinical officers demonstrated higher mean readiness scores compared to those without. Additionally, we identified statistical significant differences in mean readiness scores based on the facility cadre, type of services offered (outpatient only or both outpatient and inpatient care), and the availability of basic amenities such as communication equipment, emergency transportation, and access to a computer. Among these factors, the presence of clinical officers emerged as an independent predictor of facility readiness, with facilities having more clinical officers associated with higher readiness scores (Table 6).

**Table 6:** Differences in mean readiness scores and independent predictors of facility readiness

Variable	Mean score of readiness	*p*-value ^b^	Coeff	95% CI	*p*-value ^a^
**Facility type**					
RRH/HCIV/HCIII	7.6 (2.7)	< 0.001**	-0.86	-3.08 to 1.35	0.43
HCII	4.5 (2.3)				
**Ownership**					
Public	6.6 (2.8)	0.34	0.99	-1.62 to 3.61	0.44
Private	7.4 (3.0)				
**Location**					
Urban/Peri-urban	6.8 (2.7)	0.96	-1.83	-3.42 to 0.23	0.02
Rural	6.9 (3.3)				
**Facility teaching status**					
Teaching facility	7.6 (2.8)	0.05	0.68	-1.14 to 2.51	0.45
Not a teaching facility	6.1 (2.8)				
**Type of service offered**					
Both out and inpatient care	7.6 (2.6)	0.00***	1.32	-0.77 to 3.43	0.21
Outpatient	4.6 (2.6)				
**Health worker cadre present**					
**Doctor**					
Yes	8.6 (2.8)	0.00***	2.12	-0.22 to 4.47	0.07
No	5.9 (2.5)				
**Clinical officer**					
Yes	7.4 (2.6)	0.00***	4.01	1.49–6.53	0.00***
No	2.8 (1.4)				
**Basic amenities at facilities**					
**Power supply**					
Available	6.1 (2.6)	0.15	-1.07	-2.75 to 0.61	0.20
Not available	7.2 (3.0)				
**Improved Water supply**					
Available	7.1 (2.9)	0.12	1.29	-0.41 to 3.01	0.13
Not available	5.3 (2.3)				
**Communication equipment**					
Available	7.6 (2.6)	0.01*	-0.32	-2.42 to 1.77	0.75
Not available	5.5 (3.0)				
**Access to computer**					
Yes	8.1 (2.8)	0.01*	1.48	-0.54 to 3.51	0.14
No	6.2 (2.8)				
**Emergency transportation**					
Available	7.7 (2.6)	0.02*	-0.87	-2.65 to 0.91	0.33
Not available	5.9 (3.0)				

^*^P < 0.05, ^**^P< 0.01, ^***^P < 0.001

^b^*P*-Value for t-test, ^a^*P*-Value for linear regression

## Discussion

This study aimed to assess the readiness of facilities to screen, diagnose, and manage substance use disorders (SUD), as well as identify predictors of readiness. Our findings revealed that only 35% of the included facilities were ready to provide SUD care. The presence of clinical officers emerged as the sole independent predictor of readiness for SUD care.

To the best of our knowledge, this study is the first of its kind to examine readiness in SUD care. The observed low level of readiness among health units is not surprising, considering the low priority given to mental health in Uganda [26]. Only 1% of the entire health budget is dedicated to mental health care, reflecting the inadequate allocation of resources. Additionally, the lack of training of primary healthcare workers in mental health care contributes to this low level of readiness [27]. Similar findings of low readiness have been reported in a nationwide study conducted in Kenya, which highlighted the challenges in managing mental health [28]. These results are also in contrast to the World Health Organization’s coverage target of achieving 50% readiness by 2023 [29]. The findings from our study underscore the urgent need to enhance the readiness of health facilities to deliver SUD services and improve overall mental health care, particularly in low-resource settings like Uganda, where the prevalence of SUD is increasing. Addressing these readiness gaps is crucial for providing adequate and comprehensive care for individuals with SUD and addressing the broader mental health challenges in the country.

The findings of our study also highlighted discrepancies in the availability of complementary tracer items. Specifically, a lower percentage of facilities had screening guidelines for substance use disorders compared to those with management guidelines. This suggests that although care guidelines may exist, many facilities do not actively screen for substance use, resulting in missed opportunities for early identification of individuals at risk of developing SUD.

Furthermore, our study revealed that most facilities had availability of substance withdrawal drugs, antidepressants, and anticonvulsants, while the presence of antipsychotic drugs was limited. This shortage of antipsychotic medications reflects a lack of confidence among patients in the healthcare system’s ability to manage mental health conditions. Other studies have shown that individuals often prefer seeking care from traditional healers, even though they seek treatment for conditions such as epilepsy in healthcare facilities [1, 30]. The availability of medicines like diazepam in our study contrasts with reports of low availability in other low- and middle-income countries (LMICs), highlighting the slow progress some countries are making in integrating mental health services into primary care in LMICs [31, 32].

Although our study found adequate access to medication for managing substance use disorders across different facility levels, including lower-level health facilities, it is important to note that the presence of medicines alone is insufficient to ensure their effective use [33, 34]. Trained health workers who can appropriately administer and monitor the use of these drugs are also crucial in ensuring their optimal utilization [1].

Maintaining an adequate number of trained health professionals is crucial for achieving a balance between human and physical resources and ensuring the effectiveness of the healthcare system. Several studies have documented that the shortage of trained staff serves as a barrier to providing quality care [35–37]. Although guidelines for managing substance use disorders were present in most facilities, the number of staff trained in screening, diagnosing, and managing SUD was limited. This finding aligns with studies conducted in Bangladesh and Nepal, which revealed that the availability of protocols or guidelines in a facility does not guarantee their implementation if employees lack the necessary training [38]. In our context, this shortage of trained staff can be attributed to the insufficient number of mental health professionals and a scarcity of students choosing to specialize in mental health care [1, 20].

Overall, these findings shed light on the gaps and challenges in the availability and utilization of essential medicines and the integration of mental health services within the primary care setting. Addressing these issues is vital to enhance the overall quality of mental health care delivery and improve outcomes for individuals with substance use disorders in LMICs.

The availability of basic diagnostic equipment, such as urine test strips, was high in nearly all facilities, while the availability of blood alcohol tests was significantly lower, particularly in certain facility levels. The disparity in availability between urine test strips and blood alcohol tests may be attributed to the lower cost of urine test strips. However, it is important to note that blood tests are generally more precise, capable of determining the exact amount of alcohol or other substances, and can detect alcohol and drug compounds themselves rather than just their metabolites [39]. These findings emphasize the need for responsible authorities and healthcare stakeholders to consider increasing the availability of blood alcohol tests at all facility levels. Additionally, exploring alternative non-invasive testing methods, such as saliva and hair tests, could also be beneficial. The lack of appropriate testing options hinders the diagnosis of cases that may not be clinically evident.

Stigma remains a significant barrier to individuals seeking help for substance use issues [40]. Creating private spaces has been shown to facilitate care-seeking for mental health conditions. Our study revealed that more than half of the facilities had private rooms available for substance use screening. However, the absence of such provisions in our context demands attention from various stakeholders who have long recognized the gap between the prevalence of mental health disorders and the limited access to treatment.

Similar to a study conducted in Tanzania on outpatient management of Diabetes mellitus [41, 42], our findings identified the presence of clinical officers as a predictor of readiness. This highlights the importance of having an adequate number of trained healthcare professionals for substance use care. The issue of understaffing in Ugandan facilities, as documented by Bintabara et al., [43] is critical in understanding the readiness to provide substance use care. Unlike other health conditions, substance use care primarily relies on the expertise of healthcare workers who conduct screening, diagnosis, and administer treatment. Therefore, addressing staffing shortages is vital to improve the overall quality and accessibility of substance use care services.

### Strengths and weaknesses of the study

This study has several strengths that contribute to its significance. Firstly, it examined all facilities across different levels of the health system in both urban and rural contexts within the district, providing a comprehensive understanding of the readiness for substance use disorder (SUD) care. Additionally, the use of an adapted WHO-SARA questionnaire, specifically tailored to focus on SUDs, ensures comparability with similar studies conducted in other low- and middle-income countries (LMICs).

However, there are certain limitations that should be considered when interpreting the findings. Firstly, the small sample size and defined geographical area restricts the generalizability of the results to other contexts, emphasizing the need for caution in drawing absolute conclusions. There is a risk for epidemiological type two errors not identifying risk factors which actually are present. Nonetheless, within the specific context of this study, valuable insights into facility readiness were obtained. Another limitation pertains to the reliance on self-reported information for certain domain tracer items, which may introduce bias. Respondents may have provided more positive perspectives of their facilities, potentially underestimating the existing gaps. Conversely, it is also possible that respondents exaggerated the gaps to draw attention.

Furthermore, being a cross-sectional study, causal relationships cannot be established, and only associations can be reported. However, this study highlighted challenges posed by a fragmented approach, regardless of existing health system structures.

## Conclusions

In this context, the readiness of health facilities to provide SUD care was found to be low, with only 35% of facilities meeting the criteria. Additionally, the availability of guidelines for screening SUD was limited, indicating a gap in proactive screening practices. While diagnostic tests for SUD, such as the Uri stick, were generally available in most facilities, few had staff trained in the diagnosis and management of SUD.

The presence of clinical officers at health facilities emerged as a significant predictor of readiness for SUD care. Therefore, to enhance facility readiness, attention should be directed towards improving staffing levels and skills for management of SUD, particularly by increasing the number of clinical officers. This can be achieved through initiatives such as providing mental health training to more staff and ensuring the availability of guidelines for SUD care at facilities. By addressing these factors, facility readiness for SUD care can be improved, leading to better identification and management of SUD cases within the healthcare system. Lastly, we hope that these findings will inform and improve service delivery and research for SUD care in low-resource settings, guiding efforts towards comprehensive and integrated approaches to address these gaps. We strongly recommend several interventions, including the enhancement of training for health facility staff in identifying and managing Substance Use Disorder (SUD) cases. Additionally, improving access to information regarding available SUD care services is essential. These measures can be tailored and effectively communicated through facility information points.

## Data Availability

The datasets generated and/or analysed during the current study are available upon request.

## Abbreviations

AUD: Alcohol Use disorders
LMICs: Low- and Middle-Income Countries
SARA: Service Availability and Readiness Assessment
SU: Substance Use
SUD: Substance Use disorders
WHO: World Health Organization
